# Dietary fat/cholesterol-sensitive PKCβ-RB signaling: Potential role in NASH/HCC axis

**DOI:** 10.18632/oncotarget.17890

**Published:** 2017-05-15

**Authors:** Wei Huang, Devina Mehta, Said Sif, Lindsey N. Kent, Samson T. Jacob, Kalpana Ghoshal, Kamal D. Mehta

**Affiliations:** ^1^ Department of Biological Chemistry and Pharmacology, Dorothy M. Davis Heart and Lung Research Institute, The Ohio State University College of Medicine, Columbus, Ohio, USA; ^2^ Department of Medicine, University of Cincinnati College of Medicine, Cincinnati, Ohio, USA; ^3^ Department of Cancer Genetics, OSU Comprehensive Cancer Center, The Ohio State University College of Medicine, Columbus, Ohio, USA

**Keywords:** dietary fat, protein kinase Cβ, retinoblastoma phosphorylation, tumor suppressor

## Abstract

Hepatocellular carcinoma (HCC) is a frequent form of cancer with a poor prognosis, and environmental factors significantly contribute to the risk. Despite knowledge that a Western-style diet is a risk factor in the development of nonalcoholic steatohepatitis (NASH) and subsequent progression to HCC, diet-induced signaling changes are not well understood. Understanding molecular mechanisms altered by diet is crucial for developing preventive and therapeutic strategies. We have previously shown that diets enriched with high-fat and high-cholesterol, shown to produce NASH and HCC, induce hepatic protein kinase C beta (PKCβ) expression in mice, and a systemic loss of PKCβ promotes hepatic cholesterol accumulation in response to this diet. Here, we sought to determine how PKCβ and diet functionally interact during the pathogenesis of NASH and how it may promote hepatic carcinogenesis. We found that diet-induced hepatic PKCβ expression is accompanied by an increase in phosphorylation of Ser780 of retinoblastoma (RB) protein. Intriguingly, PKCβ^-/-^ livers exhibited reduced *RB* protein levels despite increased transcription of the RB gene. It is also accompanied by reduced RBL-1 with no significant effect on RBL-2 protein levels. We also found reduced expression of the PKCβ in HCC compared to non-tumorous liver in human patients. These results raise an interesting possibility that diet-induced PKCβ activation represents an important mediator in the functional wiring of cholesterol metabolism and tumorigenesis through modulating stability of cell cycle-associated proteins. The potential role of PKCβ in the suppression of tumorigenesis is discussed.

## INTRODUCTION

Hepatocellular carcinoma (HCC) accounts for > 90% of all primary liver cancers and generally arises in the context of chronic liver diseases [1, 2]. Recent studies suggest that abnormal lipid metabolism may play an essential role in the development of nonalcoholic steatohepatitis (NASH) and its progression to HCC [3]. Excess dietary cholesterol has been shown to cause development of experimental NASH in different animal models, especially in the setting of a concurrent high-fat diet [4–7]. A high-fat and high-cholesterol (HFHC) diet is even shown to cause progression from simple steatosis to fibrosing steatohepatitis and HCC [7]. Furthermore, extensive dysregulation of hepatic cholesterol homeostasis leading to increased hepatic cholesterol levels has also been documented in NASH and HCC [3, 4, 8]. Dysregulation likely occurs at multiple levels, including increased hepatic levels of active sterol response element-binding protein-2 and elevated cholesterol biosynthesis, coupled with increased uptake of cholesterol-rich lipoproteins and decreased cholesterol excretion in bile as cholesterol. An aberrant feedback response to sterols appears to be a common phenomenon in cancer cells [9]. Free cholesterol accumulation in the liver is toxic and may be a trigger for progression of NASH and subsequent HCC development [3]. Excess cholesterol accumulation in cells is shown to trigger ER stress, oxidative stress, apoptosis, and inflammation, which aggravate NASH [10–13].

Our recent demonstration that an HFHC diet induces hepatic protein kinase C beta (PKCβ) expression, whereas PKCβ deficiency sensitizes mice to hepatic cholesterol accumulation, suggests that PKCβ has the potential to modulate inflammation, nonalcoholic steatohepatitis (NASH), and HCC [14–16]. It is interesting to note in this regard that loss of heterozygosity of human chromosome 16p region associated with the progression of HCC includes the PKCβ locus [17]. Recent studies have also indicated involvement of PKCβ and downstream ERK-1/2 in regulating lipid metabolism, oxidative stress, inflammation, and apoptosis, key aspects of the pathophysiology of HCC [18, 19]. PKCβ is reported to phosphorylate retinoblastoma (RB) *in vitro* [20]. The retinoblastoma (RB) gene, located at human chromosome 13q14, is also associated with development or progression of carcinomas in a large spectrum of tissues because this gene is often found to have been deleted in a variety of tumor types, including HCC [21–23]. In fact, 25–67% loss of heterozygosity on chromosome 13q was reported in HCC [24].

It is well established that the liver plays a critical role in regulating cholesterol homeostasis and is targeted by both dietary fat and cholesterol [25]. We previously assessed whether metabolic adaptation to an HFHC diet is associated with changes in hepatic PKCβ expression and also studied the consequence of PKCβ deficiency on diet-induced hepatic cholesterol homeostasis. We proposed that diet-induced PKCβ activation and dependent changes in the liver may prevent toxicity and maintains normal liver function despite repeated exposure to a toxic dose. The underlying mechanism involved upregulation of cholesterol conversion to bile salts coupled with reduction in the uptake of dietary cholesterol and biosynthesis. These results support the possibility that PKCβ is a physiological transducer of dietary lipids and plays a critical role in hepatic adaptation to dietary fats. In order to explore the potential role of the PKCβ isoform in diet-induced HCC, we examined the effects of a PKCβ deficiency on the retinoblastoma protein and family members, as well as compared PKCβ expression in HCC versus normal tissue. Identification of PKCβ as a cellular relay in diet-mediated changes in RB function can define the signaling events that could participate in oncogenesis.

## RESULTS

To test if PKCβ and RB are functionally linked, we compared kinetics of hepatic PKCβ induction relative to appearance of phospho-RB and total RB expression levels. Establishing the temporal relationship between these variables will not only allow us to more accurately define the role of PKCβ in a physiological setting but, more importantly, will help to determine whether alteration in PKCβ expression and downstream signaling is an early event in the development of tumorigenesis.

**Figure 1:**
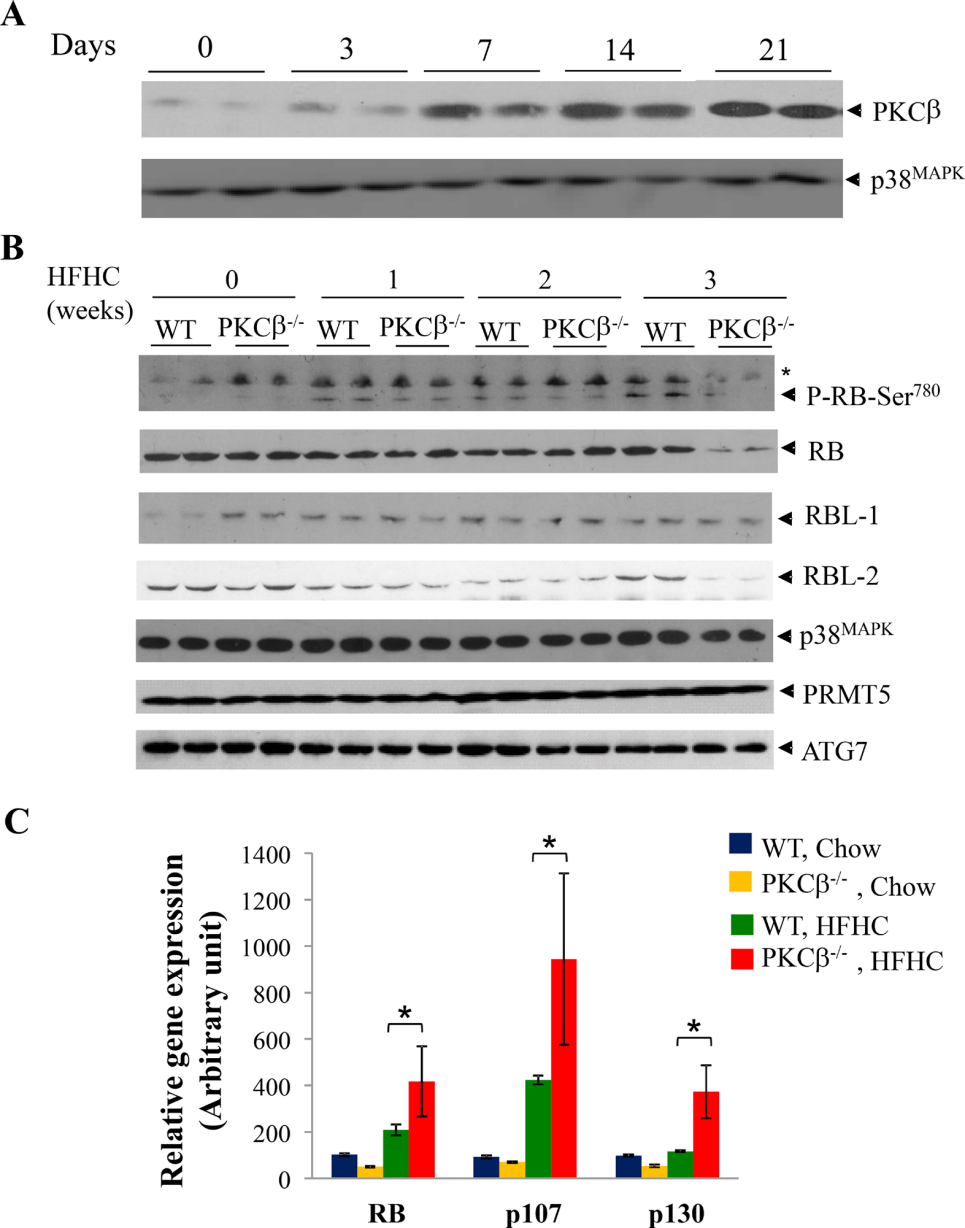
HFHC diet intake reduces RB protein content post-transcriptionally in PKCβ^-/-^ liver compared to normal WT liver (**A**) Eight-week-old WT and PKCβ^-/-^ mice (*n* = 4) were fed an HFHC diet for indicated periods and expression levels of PKCβ isoform in the pooled liver extracts (2 livers per lane) were determined by immunoblotting. (**B**) Comparison of phospho-RB, RB, p107, and p130 levels in the livers of WT and PKCβ^-/-^ mice fed an HFHC diet for the indicated periods. Equal amounts of protein were loaded per well and immunoblots were probed with antibody specific for PKCβ, RB, p107, p130, P-RB (Ser780), ATG7, PRMT5, and p38^MAPK^. (**C**) Q-RT-PCR analysis comparing relative mRNAs of RB, p107, and p130 in the pooled liver extracts of the above mice (16). Individual RNAs from the above livers were run in triplicate, and average values were calculated. Results are expressed as the mean ± SD. *, <0.05. The experiment was repeated twice with similar outcomes.

WT and PKCβ^-/-^ mice fed an HFHC diet for varying time periods were analyzed for hepatic PKCβ expression relative to phospho-RB and total RB. As shown in Figure 1A, PKCβ protein expression was markedly increased in a time-dependent manner; induction was observed at week one, peaked at two weeks, and remained elevated for at least three weeks (the last point measured). Notably, phospho-RB (Ser780) levels also showed a time-dependent increase, whereas this increase was absent in the livers of PKCβ^-/-^ mice (Figure 1B). Surprisingly, PKCβ^-/-^ mice demonstrated a marked reduction in the level of hepatic RB protein content. Total RB protein levels were decreased approximately four folds in the livers of these mice compared to controls. In contrast, expression levels of p38^MAPK^, PRMT7 and ATG7, and another family member, RBL-1, remained unaltered, whereas RBL-2 also showed a decrease in total levels in diet-fed PKCβ^-/-^ livers. A transient decrease in expression of both RBL-1 and RBL-2 was also observed in both genotypes in livers of mice fed HFHC diet for 2 weeks.

We also used quantitative RT-PCR to determine whether decreased levels of RB correlated with changes in transcript levels. When compared to control livers from mice fed an HFHC diet, RB transcript levels were increased by ∼2-fold. Likewise, an increase in mRNA levels of RBL-1 (> 2.5-fold) and RBL-2 (∼2-fold) were also noticed, possibly due to negative self-regulation and suppression of other family members by RB (Figure 1C) [26].

**Figure 2:**
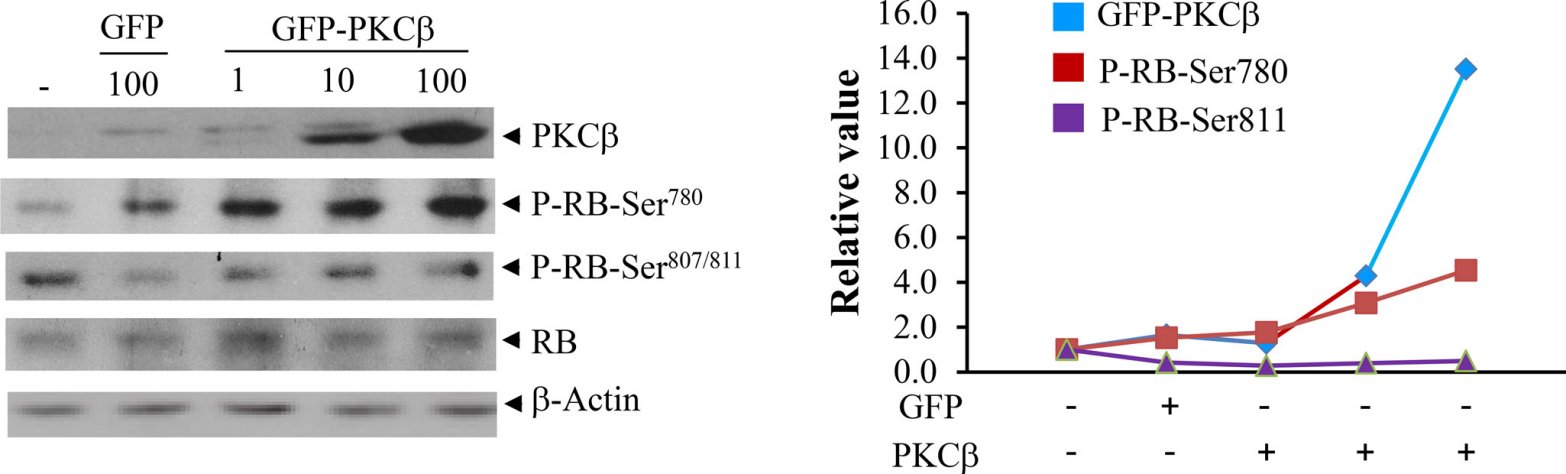
Reintroduction of human PKCβ in cultured cells restores RB (Ser780) phosphorylation with no significant effect on its abundance PKCβ^-/-^ hepatocytes were isolated from twelve-week-old C57BL/6J male mice as described previously (15). The recombinant adenovirus expressing green fluorescent protein GFP and GFP-PKCβ were used to transiently overexpress PKCβ as described previously (15, 29). After 36 h infection with 1 to 100 multiplicity of infection (MOI), cells were harvested with ice-cold PBS. Immunoblotting were used to compare phospho- and total RB levels. Quantitation of bands is shown in the right panel. The results shown are representative of at least three separate experiments. The detection of a weak band in the first two lanes possibly reflects slight cross-reactivity of the PKCβ antibody with other endogenous PKC isoforms, particularly PKCδ.

To attribute increased RB phosphorylation specifically to PKCβ, we transiently transfected PKCβ^-/-^ hepatocytes, and reintroduction of PKCβ specifically resulted in increased in phospho-RB (Ser780) levels, further supporting that PKCβ phosphorylates RB specifically at Ser780 without affecting phosphorylation at other residues (Ser807 and Ser811) (Figure 2). Notably, an increase in Ser780 phosphorylation is not accompanied by an increase in RB protein levels in cultured hepatocytes, suggesting that participation of additional diet-responsive signaling pathways dictates RB protein level.

**Figure 3:**
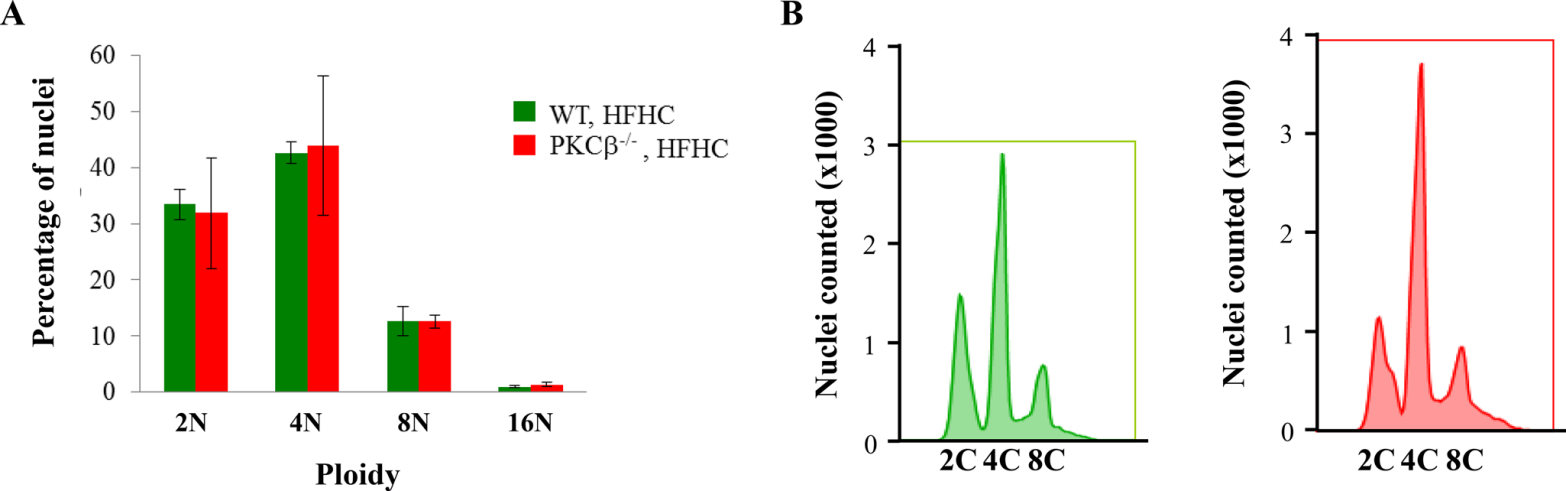
PKCβ deficiency does not increase ploidy in hepatocytes (**A**) Quantification of ploidy by FACS of hepatocyte populations from control and PKCβ^-/-^ livers (*n* = 5) after feeding an HFHC diet for three weeks. Results are expressed as the mean ± SD. * < 0.05. (**B**) Representative fluorescence-activated cell sorting profiles of propidium iodide-stained liver nuclei from the above mice. This experiment was repeated twice with similar outcomes

The global conformational changes that occur in RB upon phosphorylation are site-specific and remarkably diverse, providing a mechanism through which varying phosphorylation events can code for different functional outputs [27]. The role of S780 is presently not clear, but a previous study has reported that RB deficiency in adult mice for five to six months led to an increase in ploidy of hepatocytes [28]. In this study, loss of RB led to decreased numbers of 4n hepatocytes and a concomitant increase of 8n and 16n cells. To investigate the potential effects of PKCβ deficiency on the DNA content of hepatocytes in preneoplastic livers, we conducted flow cytometric analysis on hepatocytes obtained from WT and PKCβ^-/-^ mice fed an HFHC diet for three weeks. This analysis showed that deficiency of PKCβ had no impact on ploidy in hepatocytes (Figure 3). These observations suggest that loss of RB for a shorter period may be less influential in perturbing DNA content in adult hepatocytes, and a longer RB deficiency is needed to see any measurable effects.

**Figure 4:**
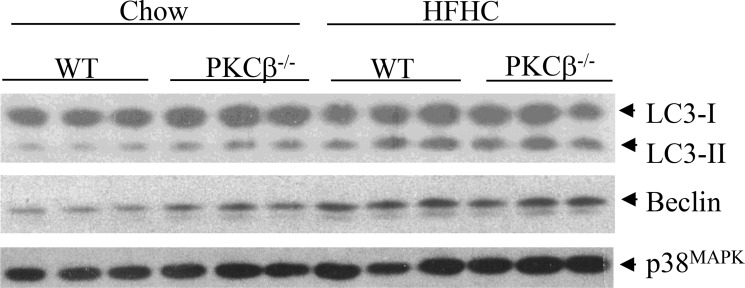
Comparison of hepatic autophagy levels between genotypes WT and PKCβ^-/-^ mice (*n* = 3) fed an HFHC diet for two weeks were sacrificed, and liver protein extracts were analyzed for the levels of autophagy markers. LC3-II accumulation was quantified by LC3-II/LC3-I ratio.

Autophagy is another protective mechanism that renders cells viable under stressful conditions. Emerging evidence suggests that this cellular process is also a tumor suppressor pathway [29]. We have previously shown that PKCβ deficiency, like RB, triggers autophagy [30, 31]. We therefore measured conversion of microtubule-associated protein LC3-I to LC3-II, a biochemical marker of autophagy that correlates with the formation of autophagosomes. As expected from our previous study, immunoblotting revealed a greater accumulation of LC3-II in the liver of PKCβ^-/-^ mice compared to WT fed a chow diet, but upon feeding an HFHC diet, the observed differences in autophagy disappeared (Figure 4). Likewise, expression of Beclin-1, a key activator of the pathway, is higher in PKCβ^-/-^ livers compared to control livers of chow-fed mice, and there is no difference in its protein levels between genotypes under dietary HFHC conditions. These results reinforce the concept that nutrient status and metabolic stressors can influence the autophagic response via signaling networks that often, but not exclusively, converge at PKCβ and RB, and participation of additional pathways potentially influence overall dietary modulation of autophagy induction. It is not surprising because autophagy is a complex process that requires a major degree of coordination among several sensors interacting with the autophagic machinery to detect fluctuations in key metabolic parameters [31].

**Figure 5:**
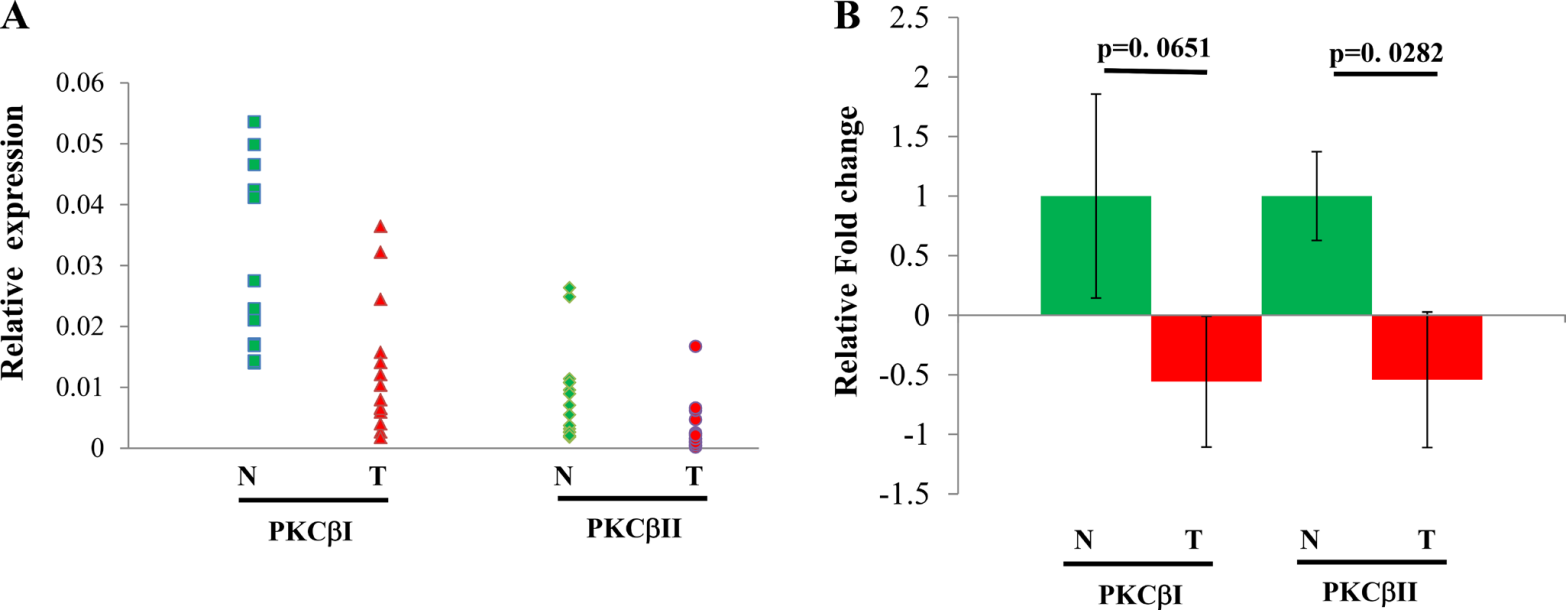
Individual (**A**) and combined (**B**) expression analyses of PKCβ isoforms in fifteen pairs of HCC and normal liver tissues by q-PCR. Total RNA from individual samples were subjected to real-time RT-PCR analysis to detect the mRNA expression of PKCβ-I and PKCβ-II. β-Actin was used as an internal control to normalize the samples. Data was analyzed using the delta delta ct method. The results were shown as mean ± SEM. All statistical analyses were performed by Student’s *t*-test or ANOVA in Excel; *p* < 0.05 was considered statistically significant. *< 0.05.

We also analyzed expression of both human splicing variants PKCβI and PKCβII in fifteen pairs of HCC and their paired nearby non-cancerous livers. The results are shown in Figure 5. The normal liver showed a homogenous expression pattern in comparison with the heterogeneous expression feature of HCC, which includes increased or decreased PKCβ mRNA in HCCs compared to control livers. We found that reduction and elevation of PKCβ was detected in 87% and 13% of HCCs examined, respectively. Interestingly, unlike PKCβI, decrease in PKCβII mRNA levels in HCCs compared to normal tissue was statistically significant (Figure 5). In addition, we compared the protein expression of PKCβII in the above samples. Again, expression was significantly reduced in the HCC livers (*p* < 0.028) when compared with normal distant and normal adjacent liver tissue (results not shown). Our data indicate that there is a downregulation of PKCβII in HCC.

## DISCUSSION

Dietary lipids are important regulators of cellular proliferation, not just as fuel substrates but also as specific modulators of the production and function of cell cycle-associated proteins. Results presented here support a role for the PKCβ regulatory network in hepatic adaptiveness to dietary fat and cholesterol. The loss of this protein may lead to the development or progression of diet-induced HCC, thus linking dietary intake of lipids with oncogenesis.

The molecular pathogenesis of HCC is complex and involves abnormal clonal expansion of dysplastic hepatocytes, anti-apoptotic signaling, and stimulation of cholesterol biosynthesis and uptake of dietary cholesterol [1, 32]. Following acute or chronic liver damage, quiescent hepatocytes enter the cell cycle and divide to restore the functional capacity of the liver. Precise control over hepatocyte proliferation is critical for the suppression of tumorigenesis in the liver, as chronic liver damage and corresponding cycles of regeneration are known to fuel the development of HCC [23]. There is clear evidence that RB pathways are involved in regulating hepatocyte proliferation. Correspondingly, the RB pathway is disrupted with high frequency in HCC [24]. Our observations suggest a role for PKCβ in the regulatory network controlling phosphorylation and stability of hepatic RB and another family member. This finding suggests that PKCβ may have some interesting biological consequences that contribute to liver tumorigenesis. Reduced PKCβ expression in a majority of our HCC samples supports a key role for this kinase as a tumor suppressor protein whose loss may predispose to tumor development. It is interesting to note in this regard that genetic alterations suppressing PKCβ activity or expression have recently been reported in a variety of cancer cell lines and in primary tumors [33, 34]. On the other hand, PKCβ overexpression observed in few HCC samples may be a programmed protective response of the organism to uncontrolled proliferation. This hypothesis is supported by a similar finding where overexpression of other tumor suppressor genes, such as *RB* and *P73*, has been detected in HCCs [35–37]. It is also possible that PKCβ, like P53 and P73, can increase its own expression in response to oncogenic activation or hypoxia or growth factor depletion/overexpression [38–40]. Individual genetic variations cannot account for heterogeneity of PKCβ expression patterns because cancer and noncancerous tissues from the same patients were examined in this study. However, it is possible that the heterogeneity of PKCβ expression is a direct result of the complexity of hepatocarcinogenesis in HCCs [41]. An important issue that might contribute to above results is the heterogeneity of HCC due to different etiologies because HCC generally arises in the context of chronic liver diseases, including chronic viral hepatitis, alcohol-induced liver injury, or other metabolic, dietary, or toxic factors such as fatty liver disease or aflatoxin ingestion. Although most of the patients had metabolic syndrome, more studies will be required to confirm and extend these observations; nevertheless, alteration in PKCβ expression implies that pharmacologic intervention of PKCβ may have broad therapeutic utility.

The exact mechanism by which PKCβ degrades RB is unknown. Multisite phosphorylation modulates the function of RB, and it has been proposed to act as a code in which discrete phosphorylation control specific activities [27]. Although the regulation of RB by phosphorylation has been extensively studied by multiple kinases, the role of phosphorylation in dictating proteasome-mediated RB protein degradation is largely unknown. Several lines of evidence indicate that RB phosphorylation and degradation are functionally linked [42, 43]. For example, gankyrin is shown to interact and stimulate RB degradation by increasing phosphorylation of specific residues in RB protein [44, 45]. Likewise, hypophosphorylated RB interacts with lamin A/C, and in the absence of lamin A/C, RB is degraded in a proteasome-dependent manner, suggesting that A-type lamins protect RB from degradation [46–48]. Given that RB undergoes enhanced degradation in a diet-fed PKCβ^-/-^ liver, it will be interesting to examine whether PKCβ directly or through downstream signal modulates RB interaction and degradation [49]. S780, implicated in cell cycle regulation, is located in a C-terminal tail of the pocket domain [50]. One possibility is that S780 phosphorylation of RB regulates phosphorylation/acetylation/ubiquitinylation/ methylation/SUMOlyation at other sites and modulates intra- and/or intermolecular association. Gaining further insight into the role of specific nutrients and PKCβ in the control of RB post-translational modifications may improve our ability to manipulate cellular proliferation.

In summary, our results uncover a unique and novel metabolic regulatory axis that couples dietary fat/cholesterol to oncogenesis, and PKCβ activation may be the initial event in delaying dietary fat-induced HCC development and therefore may be an attractive therapeutic target for preventing its development.

## MATERIALS AND METHODS

### Animals and diets

HFHC diet studies with PKCβ^-/-^ mice have been described previously [14, 15]. All mice were housed in a temperature-controlled room (22°C) with a 12 h light-12 h dark cycle, and maintained on a standard rodent diet (7912 rodent chow; Harland Tekland, WI). Eight-week-old WT and PKCβ^-/-^ mice were fed an HFHC diet (Teklad TD-10014; Harland Tekland, WI) containing (by weight) 15% anhydrous milk fat, 1% cholesterol, and 0.5% cholate for indicated period. Mice were fed *ad libitum* with free access to water. Unless indicated, all experiments were performed on non-fasted male mice and mice were euthanized in the morning. All procedures and experiments on mice were approved by Institution Animal Care and Use Committee of the Ohio State University.

### Western blotting

Liver were homogenized at 4°C by pestle in the buffer as previously described [14, 15]. Equal amounts of protein were run in 10% or 12% SDS-PAGE, transferred to nitrocellulose membranes and blocked in Tris-buffered saline with 0.1% Tween 20 containing 5% nonfat dry milk or BSA for 1 h at room temperature. Blots were incubated overnight at 4°C with following primary antibodies: PKCβ, total p38^MAPK^ and total ERK (Santa Cruz Biotechnology); β-Actin, p106, p130, LC3 (Sigma, MO); RB, P-RB, PRMT5, P-ERK (Cell Signaling Biotechnology).

### Isolation and culture of mouse hepatocytes

Mouse hepatocytes were isolated from 12 week-old C57BL/6J male mice as described previously [15]. Briefly, the liver was removed, and livers capsule were peeled off. Hepatocytes were dispersed by mechanical dissociation, and filtered through sterile100 mM-mesh nylon. The hepatocytes were washed twice in ice-cold Dulbecco’s modified Eagle’s medium (DMEM, Cellgro, Mediatech) supplemented with 10% FBS (Atlanta Biologicals, GA), penicillin/streptomycin (BioWhittaker, Lonza), insulin at 10 nM and Dexamethasone at 100 nM and resuspended in the same medium, then seeded at a density of 0.3 × 10^6^ cells per well in collagen-coated (collagen I, rat tail) 6-well plate. After 2 h incubation, the culture medium was changed without insulin. The cell culture was continued for additional 4 h before the indicated experiments.

### Infection of primary mouse hepatocytes with Ad-GFP or Ad-PKCβ

The recombinant adenovirus expressing green fluorescent protein GFP and GFP-PKCβ were used to overexpress PKCβ as described previously [15]. After 36 h infection with 1 to 100 multiplicity of infection (MOI), cells were harvested with ice-cold PBS. After spin, cells pellet were resuspended in cell lysis buffer (20 mM Tris buffer, 2 mM EDTA, 2mM EGTA, 0.1% SDS, proteinase and phosphatase inhibitors) for 30 min on ice, and sonicated briefly. The supernatant was collected after centrifugation. The protein assay was carried out before loading onto SDS-PAGE.

### Gene expression profiles

Total RNA preparation, first-strand cDNA synthesis, and the transcript levels for indicated genes were quantified as described previously [15]. Reactions were normalized to Gadph expression using the DDCt method. Relative mRNA expression was expressed as fold change over the control mice. All samples were run in triplicate, and average values were calculated.

### Flow cytometric analysis

Nuclei suspensions were obtained from frozen liver tissue as described previously [51]. Total DNA content of a minimum of 40,000 nuclei per liver sample was analyzed by the OSUCCC Analytical Cytometry Shared Resource using an LSRII (BD Biosciences). Cell cycle profiles were generated using FlowJo (Tree Star).

### Human HCC specimens

Primary human hepatocellular tumor and normal liver samples were obtained from the Cooperative Human Tissue Network at the Ohio State University James Cancer Hospital. Tissue specimens were procured in accordance with The Ohio State University Cancer Internal Review Board guidelines. The detailed information of patients is described previously [52].

### Statistical analysis

Data are presented as the mean+SE or mean+SD. To determine *p* values, two-tailed Student’s *t* tests were performed (unless otherwise indicated). *P* values indicate the level of statistical significance and *P* < 0.05 was considered statistically significant.
